# Model‐Inversion‐Resistant Physical Unclonable Neural Network Using Vertical NAND Flash Memory

**DOI:** 10.1002/advs.74517

**Published:** 2026-02-27

**Authors:** Sung‐Ho Park, Ryun‐Han Koo, Jonghyun Ko, Jiseong Im, Yeongheon Yang, Mingyun Oh, Dongbeen Shin, Gyuweon Jung, Jong‐Ho Lee

**Affiliations:** ^1^ Department of Electrical and Computer Engineering and Inter‐university Semiconductor Research Center Seoul National University Seoul Republic of Korea; ^2^ Research and Development Division SK hynix Inc. Icheon Republic of Korea

**Keywords:** neural network, security, V‐NAND flash memory

## Abstract

The growing use of neural networks in privacy‐sensitive applications necessitates architectures that inherently protect both data and model integrity. We present a model‐inversion‐resistant physical unclonable neural network (PUNN) implemented on commercial vertical NAND (V‐NAND) flash memory. A physical unclonable layer generated through weak gate‐induced drain‐leakage erase exploits intrinsic device‐level variations to create chip‐unique conductance patterns that are concealable and unreproducible. Training is achieved using the forward‐forward (FF) algorithm, which eliminates the need for backward propagation and is fully compatible with the common‐source‐line structure of V‐NAND arrays. The resulting V‐NAND FF‐PUNN demonstrates hardware‐rooted resistance to model‐cloning and model‐inversion attacks, maintaining high accuracy under forward‐only learning. When the trained network weights are transferred to another chip, inference accuracy collapses due to chip‐specific randomness, confirming intrinsic non‐clonability. Furthermore, when applied to the MIT‐BIH electrocardiogram dataset, the system achieves competitive classification accuracy on real health data while entirely blocking data reconstruction by model‐inversion. This work establishes a scalable framework for secure, energy‐efficient, and privacy‐preserving neural computing directly on commercial flash memory.

## Graphical abstract

Schematic and key features of the proposed forward‐forward physical unclonable neural network (FF‐PUNN), incorporating a concealable physical unclonable function (PUF) layer and forward‐forward (FF) learning.

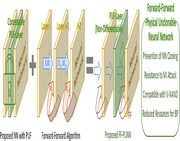

## 1 Introduction

**FIGURE 1:**
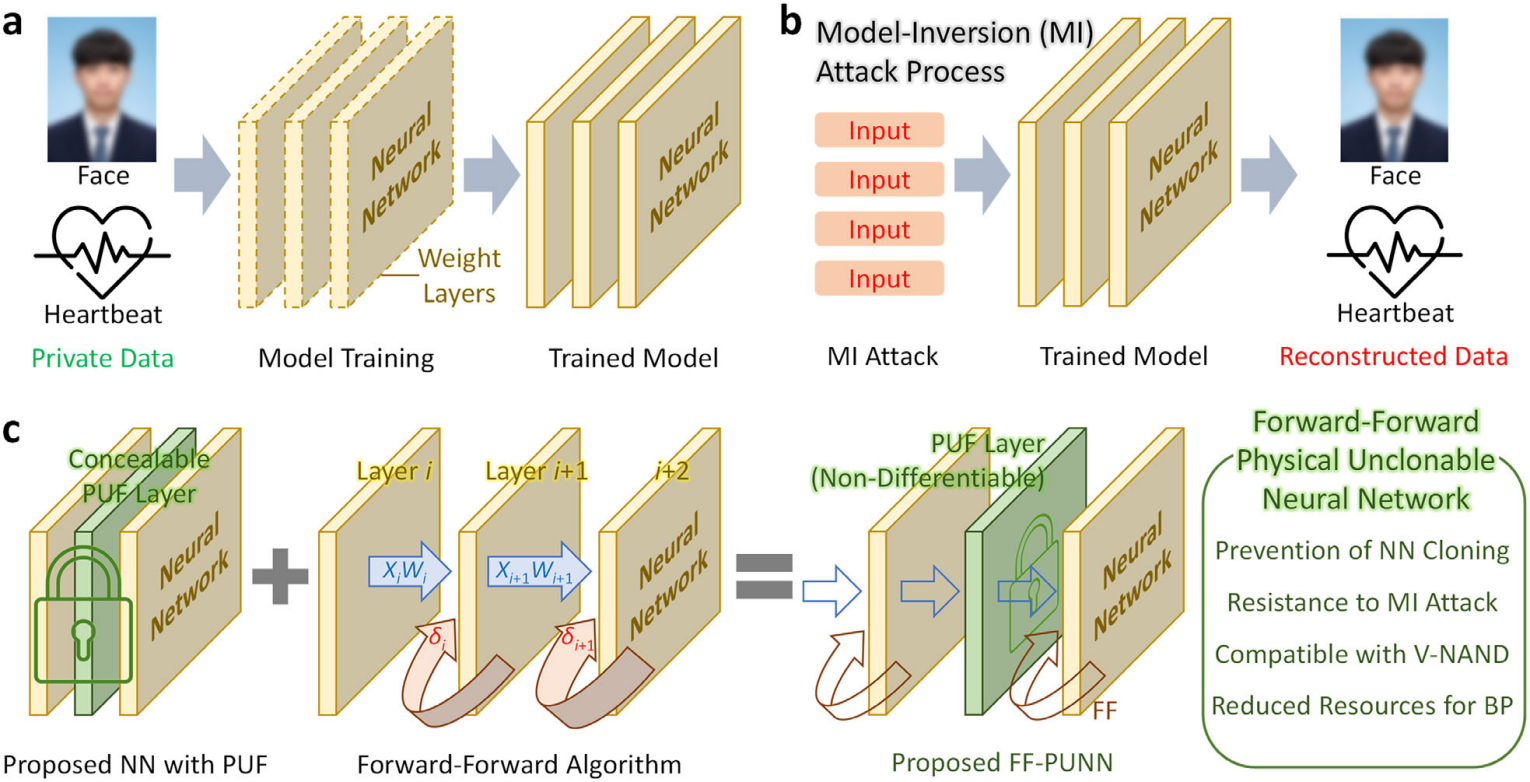
Concept and operation of the proposed Forward‐Forward physical unclonable neural network (FF‐PUNN). (a) Conventional neural network (NN) training process. (b) Model‐inversion attack process, where an adversary repeatedly queries the trained NN with arbitrary inputs to reconstruct private data. (c) Schematic and key features of the proposed FF‐PUNN, incorporating a concealable PUF layer and forward‐forward (FF) learning.

The widespread deployment of artificial intelligence (AI) in areas such as medical diagnostics, financial analytics, and biometric identification has made the protection of neural‐network (NN) models and training data a critical concern [1, 2, 3]. Modern deep networks often rely on vast datasets containing private information, and once trained, the learned parameters implicitly encode sensitive data patterns [1, 2]. This vulnerability gives rise to model‐inversion (MI) attacks, in which adversaries exploit the model outputs or accessible weights to iteratively reconstruct the original training data [4, 5, 6, 7]. As illustrated in Figure 1a,b, a conventional NN can be queried repeatedly with arbitrary inputs, enabling the attacker to approximate the data distribution that produced the model. Such attacks can reveal confidential information—such as a patient's medical image or a facial portrait—posing serious privacy risks.

Another major threat is model‐cloning, where a trained network's parameters are copied and redeployed on a different hardware platform [8, 9]. Because the original training process demands extensive computational resources and time, unauthorized replication directly undermines the intellectual property value of the trained model. These challenges—data privacy and model ownership—underscore the need for neural architectures that embed hardware‐level protection mechanisms, rather than relying solely on software encryption or access control.

To address these issues, we propose a model‐inversion‐resistant physical unclonable neural network (PUNN) implemented on vertical NAND (V‐NAND) flash memory. In this architecture, one hidden layer is replaced by a physical unclonable layer (PUL) that exploits intrinsic physical variations generated through a weak gate‐induced drain‐leakage (GIDL) erase operation. Subtle differences in the string doping depth and channel geometry create random variations in erase efficiency among V‐NAND flash cells, producing a chip‐unique conductance distribution [10]. This randomness, originating from fabrication, acts as a physical fingerprint that cannot be duplicated across chips. Importantly, the PUL remains static during training—its conductance pattern is fixed by the intrinsic physical randomness and is not updated by the learning algorithm, serving as a constant yet unique transformation within the network. Moreover, because the string doping depth remains constant after fabrication, the PUL is concealable—its random state can be hidden to a uniform programmed level during idle periods and re‐revealed only during inference by a weak GIDL erase pulse. This concealability ensures that no persistent digital copy of the random pattern exists, providing strong resistance against direct readout and side‐channel analysis [11, 12].

The overall concept of the proposed PUNN is illustrated in Figure 1c. The concealable physical unclonable function (PUF) layer is embedded between conventional computational layers, forming a hybrid system that combines algorithmic learning and hardware randomness. Since the PUL is generated directly within the flash memory itself, each chip acquires a unique internal mapping between input and output activations. When the trained weight matrices are copied onto another chip, inference behavior changes due to the new device's unique PUL pattern. This chip‐specific characteristic prevents model‐cloning and guarantees that the trained network remains functional only on the original physical hardware.

In addition, the concealed and device‐specific PUL provides strong protection against MI attacks. Since the PUL is accessible only during inference and its internal conductance distribution cannot be read or predicted, the attacker cannot estimate the relationship between model outputs and training data. Even with knowledge of surrounding layer parameters, the hidden transformation within the concealed PUL disrupts the correspondence between intermediate features and private inputs, effectively preventing data reconstruction.

Integrating such a physical layer into neural networks introduces a major challenge because it is incompatible with gradient‐based back‐propagation (BP). The PUF behaves as a black‐box function with discrete conductance states, making derivative computation impossible. To overcome this limitation, the proposed network adopts forward‐forward (FF) algorithm, in which each layer learns locally by comparing its “goodness” for positive and negative data without using backward error propagation [13, 14, 15, 16]. Unlike BP, the FF algorithm relies solely on forward signal flow, eliminating the need for bidirectional current paths or gradient computation. This property makes it highly compatible with the common‐source‐line (CSL) architecture of commercial V‐NAND flash memory [16]. In typical BP schemes, implementing reverse signal flow in a CSL array requires dividing source‐lines (SLs) or employing separate V‐NAND flash arrays, which increases complexity and energy consumption [17]. The FF algorithm enables the training process to proceed entirely through forward operations, maintaining the native CSL structure and reducing peripheral overhead.

The operation principle and advantages of the proposed forward‐forward physical unclonable neural network (FF‐PUNN) are illustrated in Figure 1c. The architecture integrates a concealable hardware layer with a gradient‐free FF learning rule, achieving both hardware security and energy efficiency in a unified framework. The FF‐PUNN retains the high density and structural compatibility of V‐NAND flash while providing built‐in resistance to weight replication and data leakage, without requiring any modification to the existing memory architecture.

Beyond its security benefits, V‐NAND flash memory is an attractive platform for neuromorphic computing [18, 19, 20, 21]. Its vertically stacked structure offers extremely high integration density, while multi‐level threshold‐voltage tuning enables analog‐weight representation for vector–matrix multiplication. The non‐volatile charge‐trap design ensures long retention and low standby power, and the mature industrial process offers scalability and cost efficiency.

In this work, we implement and evaluate the proposed FF‐PUNN using commercially fabricated V‐NAND flash memory (V‐NAND FF‐PUNN). The intrinsic device characteristics of the flash array are first measured to validate its suitability for on‐chip learning and physical‐layer security. We measure program and erase behaviors required for synaptic weight updates, as well as the PUL characteristics and reliability derived from weak GIDL erase operations. These empirical data are then used to construct and parameterize the FF‐PUNN framework. By mapping the measured conductance dynamics into the neural model, the network structure is trained, evaluated, and optimized for inference accuracy. The optimized FF‐PUNN achieves accuracy comparable to conventional BP‐based NNs while maintaining full compatibility with the unmodified V‐NAND structure. More importantly, when the trained weights are transferred to another chip, the accuracy drops drastically due to the chip‐specific randomness of the PUL, demonstrating complete resistance to model replication. Furthermore, the concealed and non‐replicable PUL distribution prevents meaningful recovery of the original training data through MI attacks, confirming that the proposed architecture provides both functional robustness and intrinsic privacy protection. Finally, to assess practical applicability on privacy‐sensitive signals, we train and evaluate the V‐NAND FF‐PUNN on the Massachusetts Institute of Technology – Beth Israel Hospital (MIT‐BIH) arrhythmia electrocardiography (ECG) dataset. FF‐PUNN attains competitive accuracy, while PUL replacement on a different chip sharply degrades performance and disrupts inversion reconstructions—demonstrating end‐to‐end feasibility, cloning resistance, and MI robustness on real health data.

## 2 Results

### 2.1 V‐NAND Flash Memory Structure and Characteristics

**FIGURE 2:**
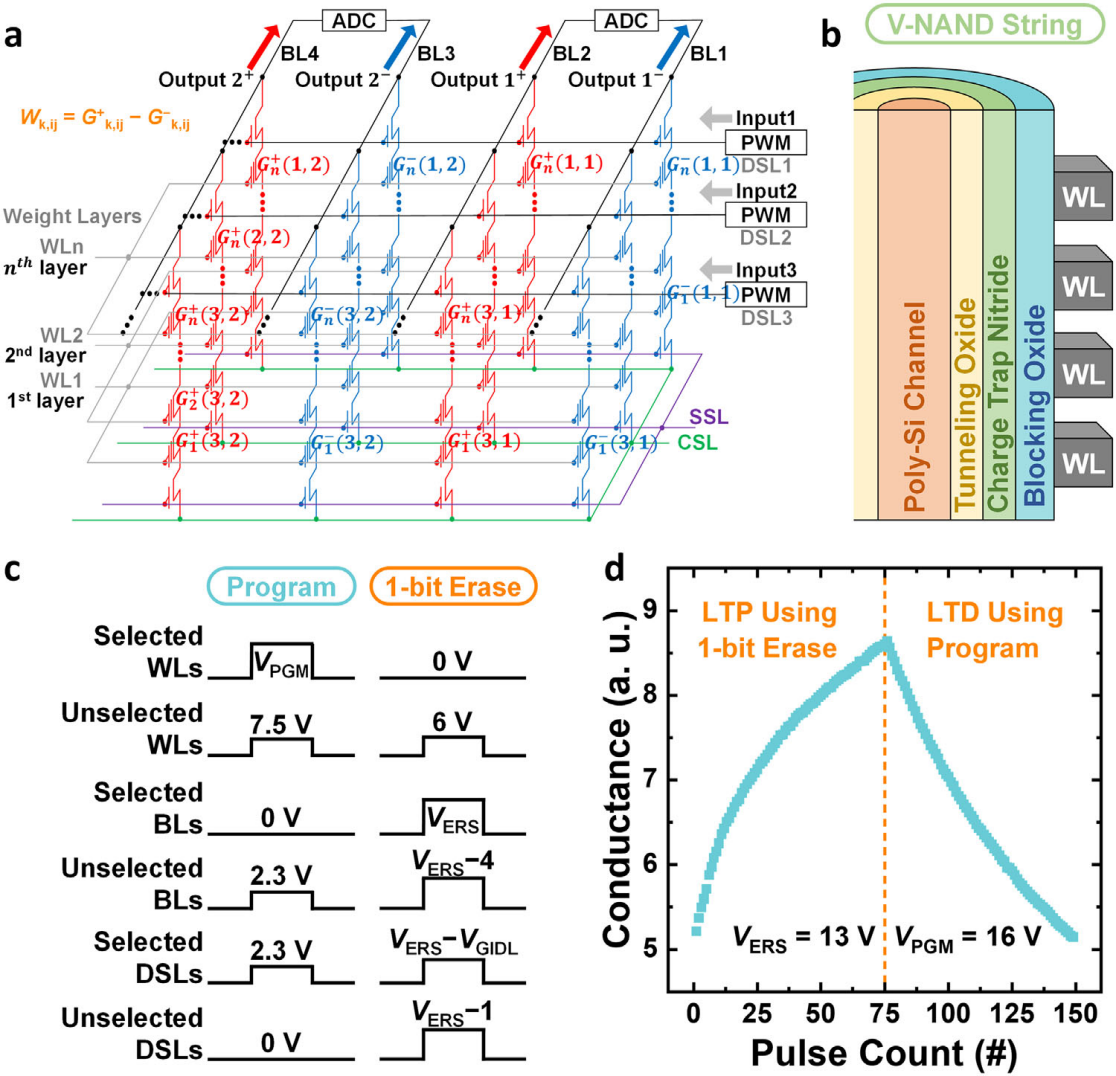
Structure and synaptic operation of V‐NAND flash memory. (a) Schematic of the V‐NAND‐based neural network architecture. (b) Cross‐sectional view of a V‐NAND string, showing the stacked gate structure. (c) Pulse scheme used for program and 1‐bit erase operations. (d) Measured long‐term potentiation (LTP) and long‐term depression (LTD) characteristics of a V‐NAND flash cell, obtained by repetitive 1‐bit erase and program pulses.

The commercial V‐NAND flash memory used in this work is fabricated by SK hynix, featuring more than 100 stacked vertical layers. Figure 2a schematically illustrates the NN architecture implemented with V‐NAND flash memory [17, 19]. Each bit‐line (BL) and drain‐select‐line (DSL) pair defines a single V‐NAND string, and the vertical stack of word‐lines (WLs) within that string determines individual flash‐cell positions. Opposite the BL and DSL across the WL layer, the source‐line (SL) and source‐select‐line (SSL) are located. Unlike the BLs, all SLs are connected together as a common‐source‐line (CSL) to reduce series resistance across the array [16]. In conventional BP‐based V‐NAND neural networks, the CSL configuration makes it impossible to apply independent input biases to individual strings. Therefore, the SLs must be separated or two independent arrays must be connected to enable BP. In the proposed FF‐PUNN, however, no BP path is required; learning proceeds solely through forward operations. This eliminates the need for array division and allows the unmodified CSL structure to be used, preserving density and energy efficiency.

The synaptic weight of each connection is stored as the conductance (*G*) of a V‐NAND flash cell. To efficiently represent both positive and negative weights, the weight value is defined as the conductance difference between two adjacent cells sharing neighboring BLs. Each WL corresponds to one weight layer in the neural network. Input signals are generated as width‐modulated pulses through pulse‐width‐modulation (PWM) circuits connected to the DSLs and are applied as read biases to selected WL [22]. During inference or training, the summed string currents are accumulated along the BLs, naturally performing vector–matrix multiplication (VMM) operations. The resulting analog current sum is then digitized by an analog‐to‐digital converter (ADC) and propagated to the next weight layer, where another VMM operation is executed.

Figure 2b shows a cross‐sectional schematic of a single V‐NAND string. The string exhibits a cylindrical vertical structure in which all WLs share a common poly‐Si channel surrounded by a charge‐trap nitride (CTN) layer, tunneling oxide, and blocking oxide. During the program operation, electrons move from the channel and become trapped in the CTN layer, increasing the threshold voltage (*V*_th_) of the selected cell [23]. Conversely, during erase, holes generated by GIDL near both string ends tunnel into the CTN layer and recombine with trapped electrons, reducing *V*_th_ [24]. Figure 2c presents the pulse scheme used for both program and erase operations [25] (Note S1).

Unlike block‐erase operations in conventional flash memory, the NN application requires individual cell control, so a 1‐bit erase scheme is adopted [26]. During program and erase operations, proper inhibition voltages are applied to unselected WLs, BLs, and DSLs to ensure that only the targeted cell experiences *V*_th_ change. For the erase operation, a GIDL voltage (*V*_GIDL_ = *V*_BL_−*V*_DSL_) is applied between the BL and DSL to generate localized holes. Because the GIDL generation rate increases with the voltage difference between BL and DSL, a higher *V*_GIDL_ produces stronger erase action. For normal 1‐bit erase operations, *V*_GIDL_ = 6 V is used to ensure complete charge removal, whereas for weak GIDL erase, which is later utilized to generate the PUL data, a reduced voltage of *V*_GIDL_ = 3 V is applied to create stable, partially erased states with distinct randomness [10].

The measured long‐term potentiation (LTP) and long‐term depression (LTD) characteristics of the commercial V‐NAND flash memory are shown in Figure 2d. These data are obtained by repeatedly applying identical sequences of 1‐bit erase and program pulses to a single cell. The conductance gradually increases during successive 1‐bit erase operations, corresponding to LTP, and decreases during successive program pulses, corresponding to LTD. The measured LTP and LTD characteristics indicate an effective analog weight resolution exceeding 6 bits. The retention stability of V‐NAND flash memory further enables reliable multi‐level storage of learned synaptic weights over long‐time scales [19, 27].

### 2.2 PUL Data Generation and Characteristics

The concealable PUF concept employed in this work is based on our previous demonstration of V‐NAND PUFs utilizing weak GIDL erase [10]. In our previous study [10], the intrinsic string‐to‐string doping‐depth variation in V‐NAND flash memory was identified as a dominant source of randomness, leading to significant differences in erase efficiency under weak GIDL bias. By intentionally applying a low GIDL voltage, partial erase occurs in some strings while others remain programmed, producing a bimodal *V*_th_ distribution across the array. This phenomenon provides a large, reproducible entropy source without any structural modification of commercial V‐NAND devices and enables concealable operation—that is, the ability to hide or reveal PUF data reversibly by simple electrical pulses.

**FIGURE 3:**
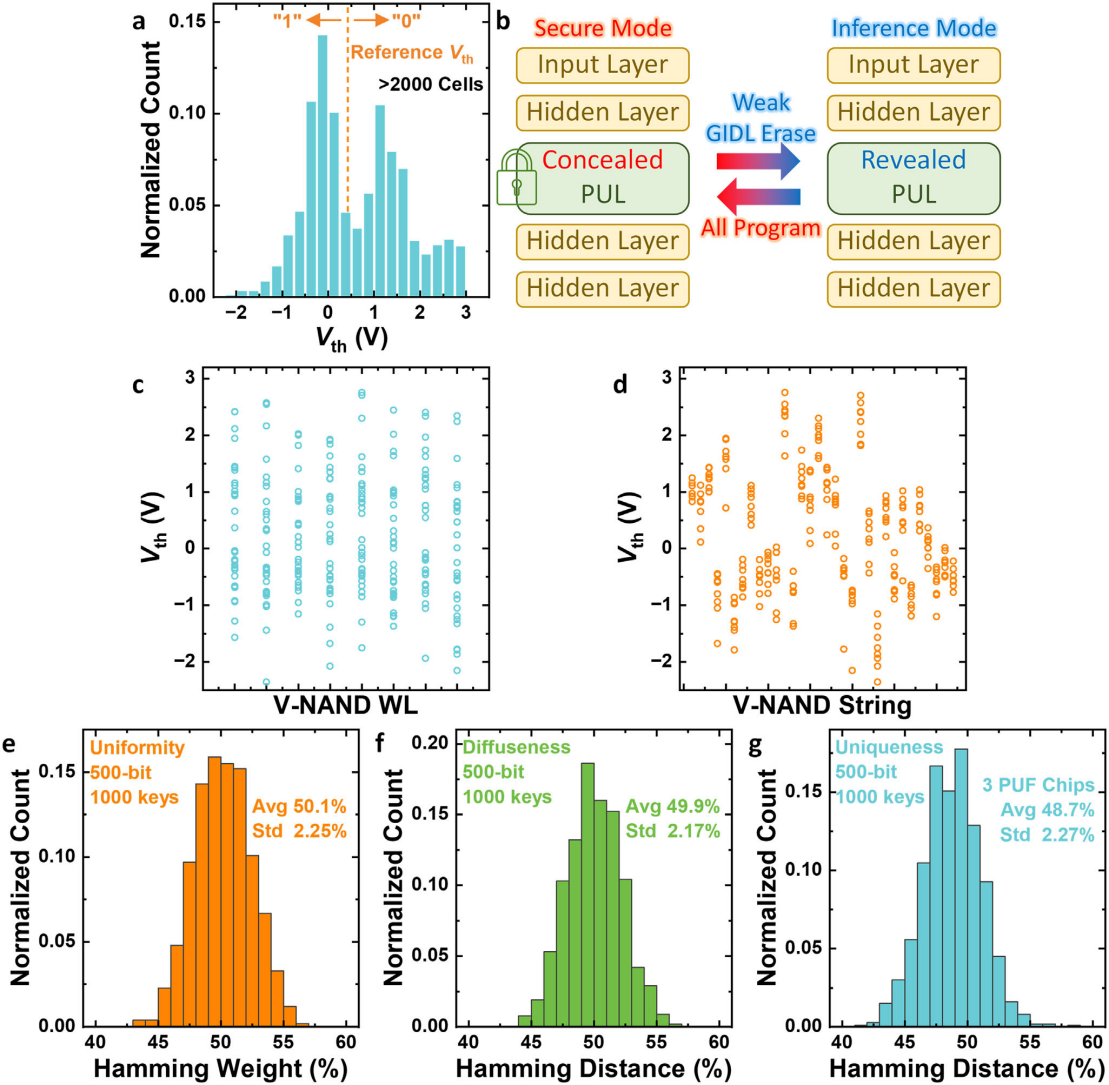
Generation and randomness of the physical unclonable layer (PUL). (a) Bimodal threshold voltage distribution obtained after weak GIDL erase (*V*_GIDL_ = 3 V). (b) Conceal/reveal operation in the proposed PUNN. During secure mode, all cells in the PUL are programmed. During inference, a weak GIDL erase reveals the PUL. (c) WL position and (d) string dependence of PUL data. (e) Uniformity, f diffuseness, and g uniqueness metrics of the constructed PUL keys.

Figure 3a presents the *V*_th_ distribution of the PUL generated through weak GIDL erase (*V*_GIDL_ = 3 V). The erased and non‐erased cells form two distinct peaks. The PUL data can be generated with a single weak GIDL erase pulse applied simultaneously to multiple cells, as illustrated in Figure 2c. The weak GIDL erase operation employed for generating the PUL is inherently based on the conventional erase mechanism of V‐NAND flash memory [19]. As such, it does not require any additional device structures, array modifications, or dedicated peripheral circuits beyond those already used for standard block‐erase operations. The PUL data can be generated using a single weak GIDL erase pulse with a duration on the order of milliseconds [28], applied only when the PUL is revealed. The reference *V*_th_ is set between these peaks to ensure a balanced bit distribution and high reliability. In this process, the input challenge determines the address of the V‐NAND flash cell to be read, and the cell's *V*_th_ is compared with the reference *V*_th_ to output a binary response of 0 or 1 as the PUF key.

The operational sequence of the proposed PUNN is shown in Figure 3b. During inference mode, a weak GIDL erase pulse is applied to the WL designated as the PUL, revealing the pre‐defined PUL data that serve as the chip‐specific randomness within the NN. Conversely, when the network is not in use, a program pulse overwrites all cells in the PUL, effectively concealing the PUL data. Because all cells are driven into the same programmed state, the unique randomness becomes inaccessible. In this concealed mode, the NN loses the distinctive mapping provided by the PUL, resulting in a dramatic degradation of inference accuracy. Consequently, both MI attacks and model‐cloning attempts fail, as the network behaves as a non‐functional model without the revealed PUL.

The physical origin of the PUL randomness is further analyzed in Figure 3c,d, which display the distribution of weak GIDL erased cells with respect to V‐NAND WL and string positions. The erase probability strongly depends on the doping depth of each string, leading to similar erase characteristics within the same string but large variations across different strings. In contrast, no clear dependency is observed along the WL direction, confirming that the variation primarily originates from string‐level process fluctuations. Since the proposed PUNN employs only a single WL as the PUL layer, one cell per string is utilized for PUL generation, maximizing the statistical independence and overall entropy of the hardware key.

Figure 3e–g summarize the statistical randomness of the constructed PUL in terms of uniformity, diffuseness, and uniqueness, which are the standard PUF metrics (Note S2) [10]. The obtained averages are 50.1%, 49.9%, and 48.7%, respectively, with standard deviations below 2.3%, all close to the ideal value of 50%. In addition, a NIST 800–22 randomness test is conducted on 10^6^ 1‐bit V‐NAND PUF responses, and all test metrics are successfully passed [10].

These results confirm that the weak GIDL–based PUL provides a well‐balanced binary distribution, high inter‐chip distinction, and consistent intra‐chip randomness. Moreover, because the string doping depth remains constant after fabrication, the PUL data can be repeatedly concealed and revealed.

### 2.3 Operation of FF‐PUNN Using PUF Layer

As discussed earlier, the bimodal *V*_th_ distribution generated by weak GIDL erase distinguishes the erased and non‐erased cells, and the resulting PUL can be utilized in two different ways. When the erased and programmed states are compared with a reference *V*_th_, the layer behaves as a PUF that outputs binary responses (0/1). Alternatively, the same *V*_th_ (or equivalently, cell conductance) values can be directly employed as analog synaptic weights for VMM operation, realizing a physical unclonable weight (PUW) layer. Hence, the proposed V‐NAND PUL can function either as a PUF‐based decision layer or as an immutable analog weight layer (PUW), depending on the network configuration.

**FIGURE 4:**
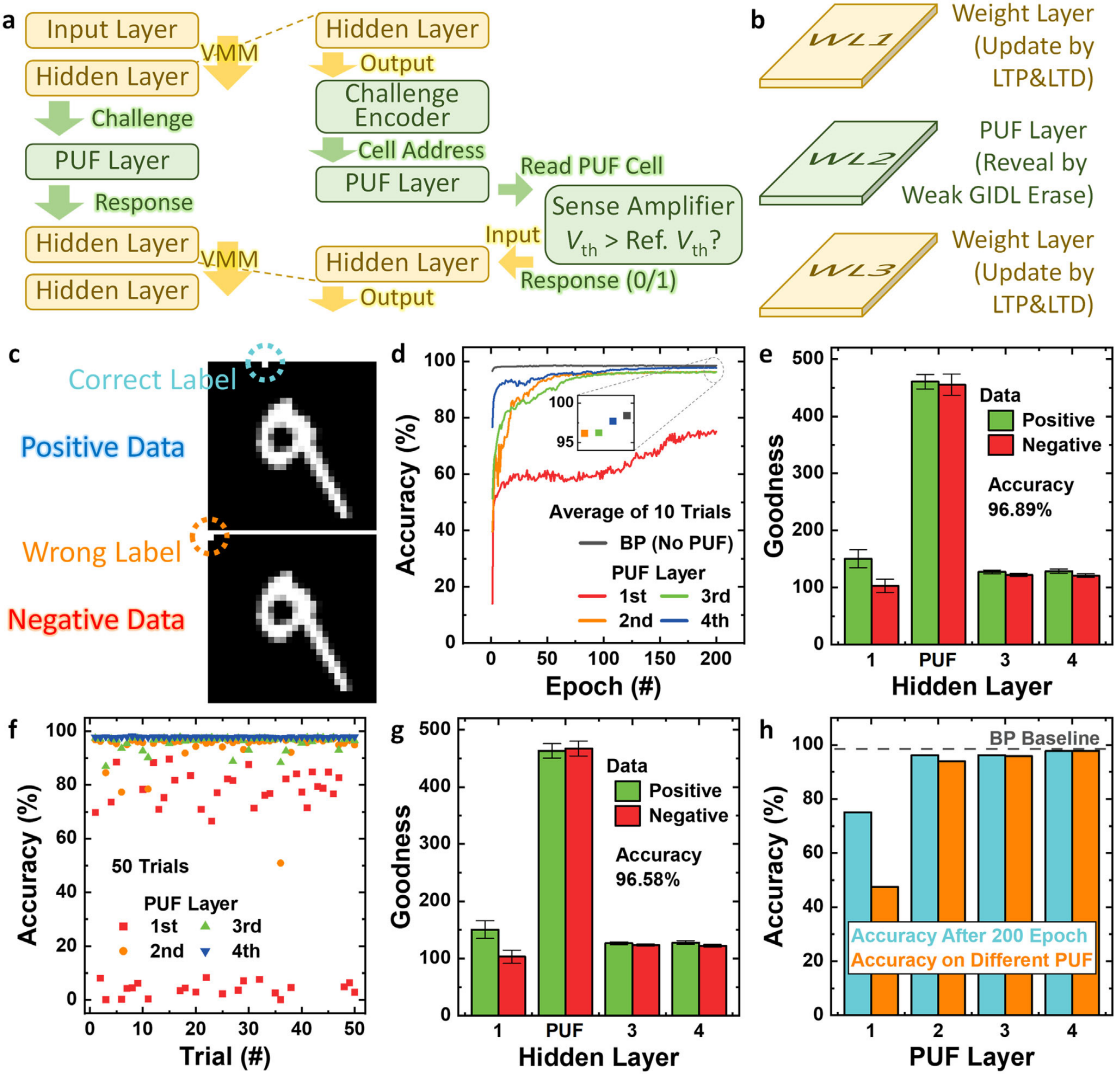
Operation and performance of the FF‐PUNN using a PUF layer. (a) Neural network architecture when the PUL is used as a PUF layer. (b) Implementation of the proposed V‐NAND‐based PUNN, where each WL functions as a weight layer, and one WL is assigned as the PUF layer. (c) Examples of positive and negative MNIST datasets used in the FF algorithm. (d) Training accuracy comparison between the baseline BP network and FF‐PUNN configurations with different PUF‐layer positions. (e) Layer‐wise goodness distribution when the second hidden layer serves as the PUF layer. The error bars indicate the standard deviation. (f) Inference accuracy after replacing the trained PUF layer with another PUF from a different chip. (g) Layer‐wise goodness after substituting the PUF chip when the second hidden layer serves as the PUF layer. h Summary of training and cross‐chip evaluation results.

Figure 4a illustrates the NN architecture when the PUL is utilized as a PUF layer. The output of the preceding hidden layer acts as the input challenge, which determines the address of the V‐NAND flash cell to be read. The corresponding cell's *V*_th_ is then compared with the reference *V*_th_, and a binary response (0 or 1) is generated and propagated to the next layer as its input. To implement this operation, the V‐NAND NN structure described in Figure 2a is augmented with a challenge encoder that converts the analog output of the previous layer into discrete cell addresses and a sense‐amplifier block that reads and compares the cell's *V*_th_. Figure 4b shows the configuration of the proposed V‐NAND PUNN. Each WL in the V‐NAND flash array serves as an independent weight layer, and the PUL is implemented by assigning one WL as the PUF layer. During the learning phase, the remaining weight layers are updated through the measured LTP and LTD characteristics, while the PUL remains fixed and is not modified by the learning algorithm. After training, the PUL can be concealed by programming all its cells to the same state, thereby erasing the underlying randomness when the network is idle.

Figure 4c shows examples of positive and negative MNIST data used in the FF algorithm. Positive data represent samples with correct one‐hot labels, while negative data are generated by assigning incorrect random labels [16]. In the FF framework, training is performed without BP. The algorithm computes the “goodness” of each hidden layer as the sum of squared neuron outputs, excluding the input layer [13]. Each layer is trained independently to increase the goodness for positive data and decrease it for negative data. In other words, FF replaces conventional forward and backward passes with two forward passes operating on different data and opposite objectives. During the positive pass, real data are processed, and the weights are updated to increase goodness in each hidden layer, whereas during the negative pass, the network uses negative data to suppress the goodness in each layer.

To evaluate the functional performance of the proposed V‐NAND FF‐PUNN, a PyTorch‐based simulation is conducted using the V‐NAND NN structure in Figure 2a and the measured device data shown in Figures 2d and 3a (Note S3). The network consists of one input layer and four hidden layers of 500 neurons each, employing the ReLU activation for rectification (Figure S1). A 7‐bit ADC is assumed at the BL output, and the first ten dimensions of each MNIST image vector are replaced by one‐hot encoded class labels—correct for positive samples and randomly permuted for negative samples, following the FF training protocol. The measured LTP/LTD and PUL conductance distributions are directly incorporated into the training model to emulate hardware behavior.

Figure 4d compares the MNIST training accuracy of a baseline BP network (without PUL) and FF‐PUNN configurations where each hidden layer is sequentially replaced with a PUF layer. The BP network without a PUL has the same structure as the FF‐PUNN except that the last hidden layer acts as the output layer. When the first hidden layer acts as the PUF layer, the accuracy degrades substantially because the original input information is immediately randomized at the beginning of the network [29]. In contrast, when the second to fourth hidden layers are used as the PUF layer, the accuracy approaches that of the BP baseline. This recovery occurs because the earlier layers remain trainable and the FF algorithm's layer‐wise “goodness” accumulation mitigates the disruption introduced by the PUF layer. Figure 4e illustrates the layer‐wise goodness when the second hidden layer is configured as the PUF layer. A clear difference in goodness between positive and negative data is observed in the first hidden layer, indicating effective feature separation during training. However, the subsequent PUF layer shows little goodness contrast between positive and negative data.

Figure 4f shows the inference accuracy when the trained PUF layer is replaced with another PUF generated from a different V‐NAND flash memory chip. This corresponds to copying the learned weights of the hidden layers to a new device while changing only the PUF layer. For the case where the first hidden layer is the PUF layer, the inference accuracy drops sharply, whereas replacement of a deeper PUF layer (second to fourth) causes negligible degradation. Figure 4g shows the layer‐wise goodness after substituting the PUF chip in the case where the second hidden layer acts as the PUF layer. As in Figure 4e, the first hidden layer still exhibits a large goodness difference between positive and negative data, while deeper layers show almost no difference, implying that the trained first layer dominates the classification. Consequently, the inference accuracy remains high even when the PUF chip is replaced, indicating limited cloning resistance. The summarized results in Figure 4h indicate that high training accuracy is achievable in the proposed FF‐PUNN, but effective security validation requires the accuracy to decrease when the PUF layer is replaced. The limited accuracy drop observed for deeper PUF layers suggests that the well‐trained first hidden layer dominates overall inference, masking the randomness introduced by subsequent PUF layers. Therefore, while using the PUF as a PUL yields high learning accuracy, its reproducibility across different PUF layers weakens cloning resistance, motivating alternative approaches such as employing the PUL as a PUW layer, discussed in the following section.

### 2.4 Reliability of FF‐PUNN Using PUW Layer

**FIGURE 5:**
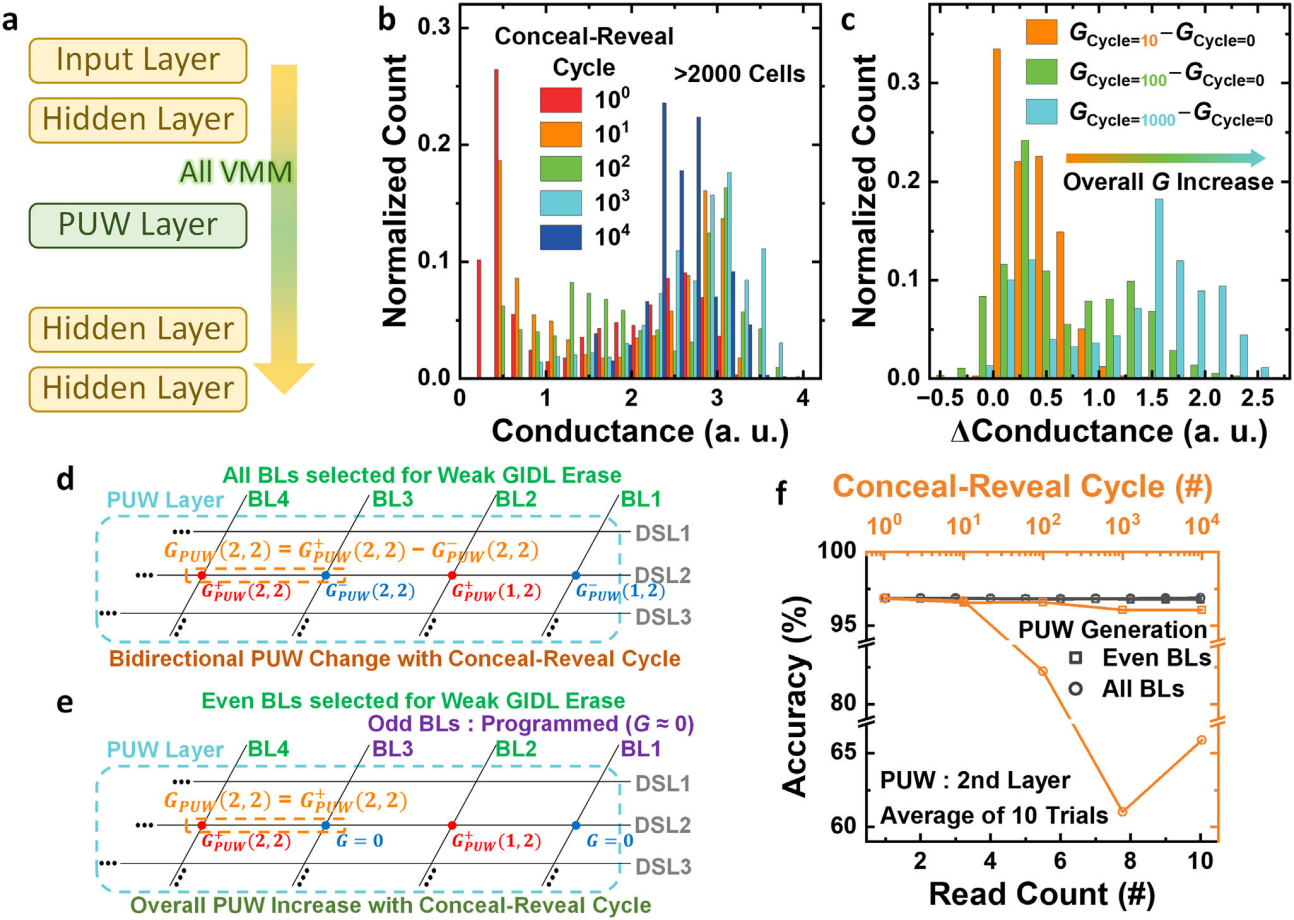
Operation and robustness of the PUW layer. (a) Network configuration using a PUW layer. (b) Conductance histograms of V‐NAND cells after repeated conceal‐reveal cycles. (c) Distribution of conductance change versus conceal‐reveal count. (d) Conventional differential‐pair weight, which fluctuate bidirectionally with conceal‐reveal cycling. (e) Proposed even‐BL‐only PUW scheme: only even‐BL cells are weakly erased to form PUW. f MNIST accuracy versus read count and cycle‐read count, comparing the all‐BL differential and even‐BL‐only PUW schemes.

In contrast to the PUF‐layer configuration described previously, the PUW layer employs the bimodal *V*_th_ (or conductance) distribution generated by weak GIDL erase directly as the synaptic weights for VMM operations. As shown in Figure 5a, the PUW layer performs the same VMM operation as other hidden layers, but—unlike them—it is not updated during training. A key distinction from the PUF‐layer scheme is that the PUW layer is differentiable, since it outputs continuous conductance values rather than binary responses. Therefore, in principle, the network can also operate under BP if the hardware structure allows it. However, as discussed earlier, the CSL configuration of commercial V‐NAND flash memory restricts the current flow required for BP, whereas the FF algorithm operates solely through forward passes without additional wiring or duplicated arrays. Consequently, FF training provides a more hardware‐compatible solution for V‐NAND PUNN.

Unlike the binary PUF layer, the PUW layer uses real‐valued conductance as weights, which makes the network more sensitive to gradual conductance variations. Because the PUL data are revealed only during active operation and concealed at other times for security, repeated conceal–reveal cycles can alter the cell conductance and degrade network accuracy. Figure 5b shows the conductance distribution of V‐NAND flash cells measured after repeated conceal–reveal operations, and Figure 5c presents the corresponding distribution of conductance changes. As the number of cycles increases, the overall conductance gradually shifts upward, which is attributed to an increase in trap density within the tunneling oxide [30]. To mitigate accuracy degradation caused by this cumulative conductance increase, the weight‐calculation method of the PUW layer can be modified.

In conventional V‐NAND NNs, as shown in Figures 2a and 5d, each synaptic weight is represented as the conductance difference between two adjacent BL cells (*G* ^+^−*G* ^−^) to encode both positive and negative values. However, under repeated conceal–reveal cycling, the conductance of all PUL cells tends to increase, leading to bidirectional fluctuations in the differential weights. Such random two‐way drift critically reduces inference accuracy. To overcome this, the PUW layer is redesigned to compute weights using only one cell per synapse, as depicted in Figure 5e. In this scheme, odd BL cells are permanently kept in the programmed (low‐*G*) state, while even BL cells alone are weakly erased to generate the PUW data. Consequently, the effective weight corresponds directly to the conductance of the even BL cell (*G* ^+^), which changes only in the positive direction with additional conceal–reveal cycles, preventing severe bidirectional degradation. Figure 5f compares the MNIST‐classification accuracy as a function of read count and conceal–reveal count for two PUW configurations: using all BL cells to compute weights by conductance difference, and using only even BL cells as proposed. For increasing read counts, both cases show stable accuracy. However, as the conceal–reveal count rises, the differential‐pair method exhibits a drastic accuracy drop due to random bidirectional weight drift. In contrast, the proposed even‐BL‐only scheme effectively suppresses accuracy loss by maintaining unidirectional weight evolution, enabling the V‐NAND PUNN to maintain stable inference accuracy up to 10^4^ conceal‐reveal cycles. The operational stability under repeated conceal‐reveal operation of the PUL is significantly higher than that reported in prior concealable PUF implementations based on emerging memory devices, such as memristors [31, 32].

### 2.5 Operation of FF‐PUNN Using PUW Layer

**FIGURE 6:**
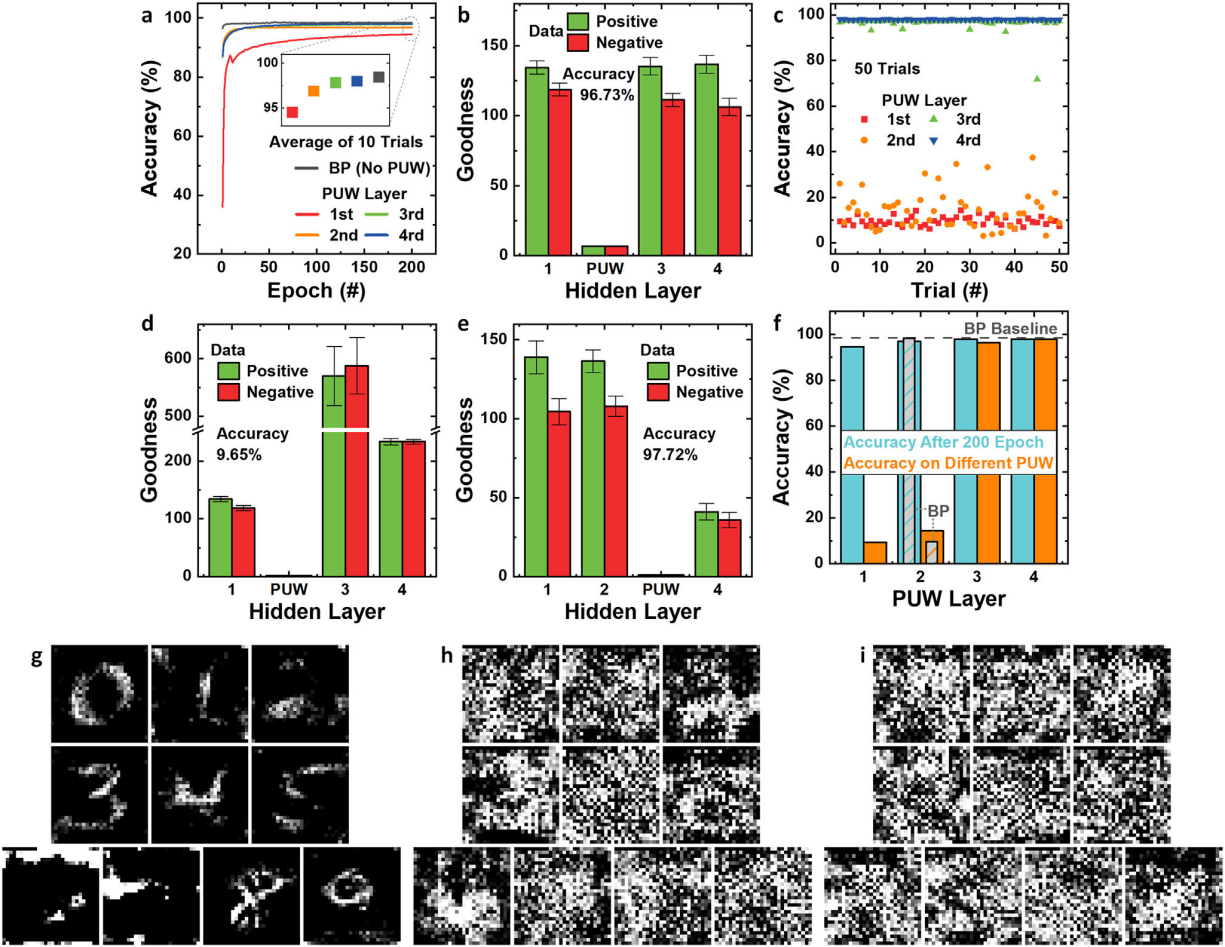
Performance and security of FF‐PUNN with a PUW layer. (a) MNIST accuracy versus PUW‐layer depth. (b) Layer‐wise goodness when the second hidden layer is the PUW. (c) Cross‐chip robustness result: accuracy after replacing only the PUW layer with another chip's PUW. The error bars indicate the standard deviation. (d) Goodness after PUW replacement for a PUW trained at the second layer and (e) third layer. (f) Summary of training and post‐replacement accuracies for each PUW depth. Hatched bars indicate BP‐trained counterparts (when hardware permits), showing similar trends. Model‐inversion attack reconstructions: (g) FF network without PUL, (h) FF‐PUNN with second‐layer PUW, and (i) when PUW replaced by a different chip's PUW.

Figure 6a reports MNIST classification accuracy when the PUW layer is placed at different depths in the network. Weak GIDL erase is applied only to cells on even BLs as shown in Figure 5e, while odd‐BL cells are kept programmed. As with the PUF layer experiments, the PUW layer placed closer to the input (front) degrades accuracy more severely than when positioned deeper in the network. This trend reflects the fact that early layers process raw input information; inserting a fixed, device‐unique weight layer too early removes or distorts low‐level features that subsequent layers cannot fully recover. Figure 6b shows the layer‐wise goodness for the case where the second hidden layer is configured as the PUW layer. The total goodness for positive data is significantly larger than that for negative data, indicating that despite the fixed, device‐specific weights in the PUW layer, the network still learns separable internal representations and attains stable classification performance when trained under the FF scheme.

To examine robustness to hardware cloning, Figure 6c presents inference accuracy when the trained model is ported to a different V‐NAND device by replacing only the PUW layer (i.e., other learnable weights are copied to the new chip while the PUW is changed). When the PUW occupies the first or second hidden layer, the accuracy drops markedly upon replacement of the PUW layer. This behavior demonstrates that the model's correct mapping from inputs to outputs depends critically on the specific physical weights of that PUW layer; changing them breaks that mapping and severely degrades performance. In contrast, if the PUW layer is placed deeper (e.g., third layer), the earlier trainable layers preserve much of the discriminative representation, and the accuracy after PUW replacement is comparatively less affected. Figure 6d,e shows the layer‐wise goodness distributions after replacing the PUW chip for the cases where the PUW was trained at the second and third hidden layers, respectively. When the second layer is the PUW, replacing it causes large shifts in the goodness patterns of downstream layers (third and fourth), which explains the large accuracy loss seen in Figure 6c. By contrast, when the third layer is the PUW, the first and second hidden layers retain their learned goodness separation between positive and negative data even after replacement, and downstream changes are smaller; hence the overall accuracy degrades much less.

Figure 6f summarizes these findings by plotting the training accuracy and the post‐replacement accuracy for each PUW placement. The key security implication — and the primary metric we therefore propose for device‐level protection — is the accuracy drop upon PUW replacement. A large drop indicates that the model is tightly coupled to the original chip's PUW data, so a cloned copy (weights copied to a different chip) fails to deliver the same functionality. For the case where the second hidden layer is the PUW, the model shows both high training accuracy and a substantial accuracy drop when the PUW is swapped out, indicating strong resistance to model‐cloning. The hatched bars in Figure 6f indicate results obtained when BP (rather than FF) is used for training; these show a similar pattern, implying that if the hardware can support BP, including a device‐unique PUW layer still yields both high accuracy and strong cloning resistance. For applications that require higher inference accuracy or involve more complex datasets, the proposed framework is not fundamentally limited to forward‐only learning. If a V‐NAND architecture with divided SLs, rather than the conventional CSL configuration, is employed, the PUW layer can be integrated with BP training.

Beyond cloning, we evaluate MI attack resilience. MI attack is conducted as activation maximization: starting from a random image, we maximize the FF goodness minus the average score of non‐target classes (Note S4) [13]. Figure 6g–i show reconstruction results from a standard MI attack applied to three configurations: (g) an FF network without any PUL/PUW, (h) an FF‐PUNN trained with the second‐layer PUW, and (i) the same as (h) but with the PUW replaced by a different chip's PUW prior to attack. In the no‐PUW baseline, many digits reconstructed by inversion are visually recognizable, demonstrating that a model exposed without device‐unique randomness can leak training‐data features. In Figure 6h, simply introducing the PUW layer substantially degrades the quality of reconstructed images; many digits become noisy and unrecognizable, although a few shapes such as ‘2’ or ‘3’ may still appear faintly. In Figure 6i, after replacing the PUW with a different chip's PUW, the inversion outputs degrade further and yield largely meaningless noise patterns — the inversion attack is effectively thwarted. It is worth noting that concealing the PUL produces an effect equivalent to replacing the PUW with a chip‐distinct PUW. When the PUL is concealed, all cells in the PUW layer are overwritten to a uniform programmed state, effectively erasing the device‐specific conductance pattern.

### 2.6 ECG Classification With V‐NAND FF‐PUNN

**FIGURE 7:**
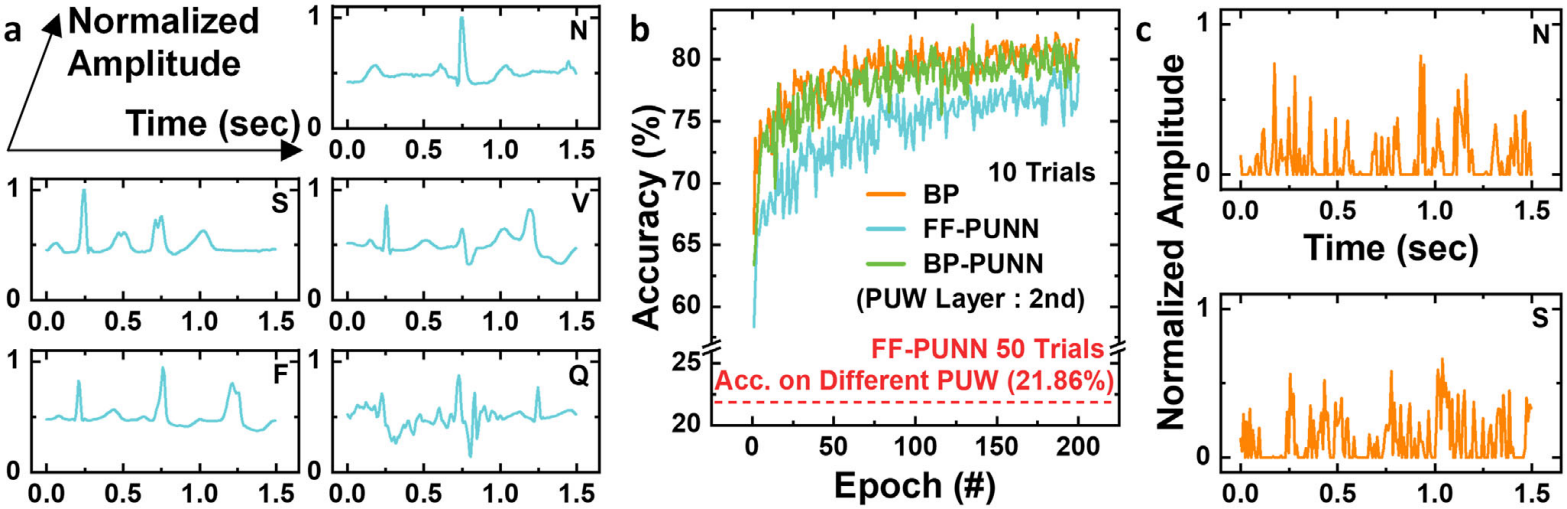
Demonstration of the proposed V‐NAND FF‐PUNN on real health data using the MIT‐BIH ECG dataset. (a) Representative ECG waveforms from the five AAMI classes used for classification. (b) Training accuracy of the V‐NAND PUNN with the PUW layer placed at the second hidden layer. The PUNN is implemented using both BP and FF. (c) Results of the MI attacks targeting the N and S classes after deploying the trained V‐NAND FF‐PUNN on a different chip.

We further validate the proposed framework on a privacy‐sensitive, real‐world health dataset: the MIT‐BIH Arrhythmia database [33]. Figure 7a illustrates representative waveforms from the five AAMI classes used in our experiments: N (Normal / non‐ectopic), S (Supraventricular ectopic), V (Ventricular ectopic), F (Fusion of ventricular and normal), and Q (Unknown / unclassifiable). Signals are preprocessed into fixed‐length segments and fed to the network as in our MNIST PUNN, with the PUL configured as a PUW layer at the second hidden layer. Figure 7b reports the classification accuracy. Using FF training, the V‐NAND FF‐PUNN achieves ∼80% test accuracy, which is modestly lower than a BP‐trained baseline but demonstrates that forward‐only learning remains effective on multi‐class ECG. The V‐NAND PUNN trained with BP achieves high accuracy comparable to the baseline. Crucially, when the trained FF‐PUNN model is deployed on a different V‐NAND chip (i.e., only the PUW layer changes), accuracy collapses to ∼20%, indicating that the decision function is tightly bound to the original device's PUL and cannot be cloned by weight copying. We also assess model‐inversion resilience. Figure 7c shows MI reconstructions targeting the N and S classes after replacing the PUW layer with a different chip. The synthesized waveforms deviate substantially from the true training exemplars in Figure 7a, lacking class‐specific morphology—evidence that the cross‐chip model offers no meaningful recovery of private ECG signals. Together, these results confirm that FF‐PUNN's chip‐specific physical mapping prevents cloning and neutralizes inversion attacks on sensitive health data when the model is copied to non‐original hardware.

## 3 Discussion

This study demonstrates that a commercially fabricated V‐NAND flash memory can serve not only as a non‐volatile synaptic medium but also as a physical security primitive for NNs. By embedding a concealable PUL and employing gradient‐free FF training, we achieve end‐to‐end protection against both model‐cloning and MI attacks without modifying the native flash architecture. The chip‐specific physical randomness guarantees that copying trained weights to another device leads to severe accuracy degradation, while the conceal–reveal operation ensures that the physical entropy remains hidden when the system is inactive.

Beyond its security functionality, the V‐NAND platform offers important practical advantages for large‐scale and deployable secure neural systems. State‐of‐the‐art V‐NAND flash memory integrates more than 400 vertically stacked WL layers [34], enabling extremely high storage density within a compact footprint. This vertical scalability allows the proposed PUNN architecture to incorporate a large number of synaptic weights and physical unclonable elements without incurring significant area overhead, underscoring the suitability of mature V‐NAND technology for secure and scalable neural‐computing hardware.

The concept was further validated using real health‐care data (MIT‐BIH ECG), confirming that the proposed architecture preserves functionality on privacy‐critical signals while blocking reconstruction of patient‐specific features. These results highlight the practical applicability of the FF‐PUNN for secure biomedical and edge‐AI hardware.

Nevertheless, the current implementation also reveals limitations intrinsic to the FF algorithm. Because FF learning updates each layer locally, its global optimization capability remains weaker than that of back‐propagation; for example, the FF‐trained network achieves only about 60% accuracy on CIFAR‐10^13^, compared with 80–90% for BP under similar conditions. Improving this performance gap will require hybrid approaches that combine local goodness‐based objectives with partial gradient propagation or meta‐learning techniques, as well as architectural regularization to better align local and global feature hierarchies.

Looking ahead, integrating optimized FF‐based training with larger‐scale V‐NAND flash memories could provide a practical path toward secure, low‐power, and privacy‐preserving neuromorphic systems that operate directly on ubiquitous flash memory hardware.

## 4 Methods

### 4.1 V‐NAND Flash Memory for Measurements

Commercial triple‐level cell (TLC) V‐NAND flash memory chips supplied by SK hynix are used in this study. The devices feature a vertical stack exceeding 100 WL layers.

### 4.2 Electrical Characterization

All device‐level characteristics are obtained using a Keysight B1500A semiconductor parameter analyzer connected to a probe station. A B2201A switching matrix is employed to accurately route the programmed voltage signals to the corresponding contact pads.

### 4.3 V‐NAND Flash Memory Operation Procedures

Standard program, read, and GIDL erase sequences are performed following a conventional pulse‐based operation scheme (Note S1) [25].

### 4.4 Machine Learning Simulation

Machine‐learning simulations for the MNIST and MIT‐BIH datasets are conducted using the PyTorch framework, incorporating the LTP/LTD characteristics, PUL behavior, and other device‐level properties measured from the V‐NAND flash memory (Note S3). All ADC operations are performed with a 7‐bit resolution.

### 4.5 Model‐Inversion Attack

For model‐inversion attack, we perform an activation‐maximization attack on the trained neural network. The attack attempts to synthesize an input image whose FF “goodness” strongly favors a specific class over the remaining classes (Note S4) [13].

## Author Contributions

S.H.P. proposed the idea, performed the experiments, and wrote the paper. R.H.K., J.I., M. O., and D.S. designed the experiments. Y.Y. and G.J. set up the experimental protocol. J.K. performed the machine learning simulation. J.H.L. supervised the project. All the authors discussed the results and commented on the manuscript.

## Conflicts of Interest

The authors declare no conflict of interest.

## Supporting information




**Supporting File**: advs74517‐sup‐0001‐SuppMat.pdf.

## Data Availability

All relevant data are available from the corresponding authors upon reasonable request.
