# Supplementary material for: Model‐Inversion‐Resistant Physical Unclonable Neural Network Using Vertical NAND Flash Memory
Source: Adv Sci (Weinh). 2026 Feb 27;13(25):e74517. doi: 10.1002/advs.74517 (PMC13137843; doi:10.1002/advs.74517)
Supplement: Supplementary file 1 — Supporting File: advs74517‐sup‐0001‐SuppMat.pdf. [file ADVS-13-e74517-s001.pdf]

Supplementary Information

Model-Inversion-Resistant Physical Unclonable Neural Network Using Vertical NAND Flash Memory

Sung-Ho Park[1], Ryun-Han Koo[1], Jonghyun Ko[1], Jiseong Im[1], Yeongheon Yang[2], Mingyun Oh[1], Dongbeen Shin[1], Gyuweon Jung[1], and Jong-Ho Lee[1*]

[1]*Department of Electrical and Computer Engineering and Inter-university Semiconductor Research Center, Seoul National University, Seoul 08826, Republic of Korea*
[2]*Research and Development Division, SK hynix Inc., Icheon 17336, Republic of Korea*

[*] Corresponding author. Tel.: +82-2-880-1727; Fax: +82-2-882-4658
E-mail address: jhl@snu.ac.kr (J.-H. Lee)

Supplementary Note 1

Data storage operation of V-NAND flash memory. The data-storage operation of V-NAND flash memory is based on two fundamental primitives, program and erase, as illustrated in Fig. 2c. Because programming is performed at the level of individual memory cells along a series-connected string, it is crucial to inhibit unintended charge injection into unselected cells. In conventional V-NAND flash memory, erasing is typically supported only in a block-erase mode, in which all cells within a block are reset simultaneously. In this work, to enable fine-grained weight updates for neural-network operations, we instead adopt a 1-bit erase scheme[1] that allows selective erasure of a single cell while preserving the state of other cells in the same block. When the 1-bit erase pulses are applied simultaneously to all WLs, BLs, and the drain-select-line (DSL) of a block, the scheme simply degenerates to a standard block erase.

For programming, a high positive bias is applied to the selected WL so that electrons are injected from the channel into the charge-trap nitride. Specifically, a program voltage V_{PGM} is applied to the target WL, which increases the potential difference between the WL and the underlying channel and thereby accelerates electron injection into the trap layer. A larger V_{PGM} results in a stronger programming effect due to the enhanced electric field across the tunnel oxide. To suppress programming of unselected cells along the string, a pass voltage (7.5 V) is applied to the unselected WLs. This pass bias raises the channel potential along the string while keeping the local channel–WL voltage of unselected cells below the programming threshold. On the BL side, the selected BL is held at 0 V to maximize the channel–WL potential difference under, whereas the unselected BLs are biased at an inhibit voltage (2.3 V)

The erase operation is implemented by exploiting gate-induced-drain-leakage (GIDL) to generate holes that neutralize the stored electrons. In contrast to programming, a positive erase voltage V_{ERS} is applied to the selected BL (i.e., to the channel region), while the selected DSL is biased at a lower voltage (V_{DSL}). The resulting voltage difference $V_{GIDL} = V_{ERS} – V_{DSL}$ creates a strong field at the drain junction, causing GIDL and generating holes in the channel. These

holes drift toward the WL and recombine with the trapped electrons in the nitride, thereby removing negative charge and lowering the cell threshold voltage. Increasing V_{GIDL} enhances the hole generation rate and thus strengthens the erase action.

For strings that are not targeted by the 1-bit erase, the unselected BL and DSL are biased at voltages 4 V and 1 V lower than V_{ERS}, respectively. These reduced voltage differences are insufficient to trigger GIDL, effectively suppressing hole generation in unselected strings. The selected WL is held at 0 V to maximize the vertical field for hole transport from the channel to the gate, while all unselected WLs are biased at 6 V. This high WL bias keeps their potential close to the channel level, minimizing the channel–WL potential difference and preventing any appreciable change in the threshold voltage of unselected cells. As V_{ERS} increases, the channel potential rises and the erase efficiency improves.

Supplementary Note 2

Security characteristics of the V-NAND PUF. The security properties of the proposed V-NAND PUF were evaluated in terms of uniformity, diffuseness, and uniqueness of the generated PUF keys (Fig. 3)[2]. Three independent PUF chips were implemented, and 1000 PUF keys were generated from each chip, with each key composed of 500 bits.

Uniformity represents the balance between '0' and '1' within a single PUF response; the ideal case is 50%, meaning an equal number of 0s and 1s.

Diffuseness was quantified using the intra-chip Hamming distance (intra-HD). The Hamming distance (HD) measures the number of bit positions at which two keys of the same length differ. An HD ratio of 50% is considered ideal, whereas 0% indicates that two keys are identical and 100% means that one key is the bitwise complement of the other. The intra-HD is obtained by comparing keys generated from the same PUF under different challenges.

Uniqueness was assessed from the inter-chip Hamming distance (inter-HD), calculated by comparing keys produced by different PUF chips under the same challenge. An ideal uniqueness of 50% implies that there is no correlation between responses from different PUF instances.

For the fabricated V-NAND PUFs, the distributions of uniformity, intra-HD, and inter-HD follow Gaussian-like profiles centered around the ideal value of 50%, confirming that the proposed structure achieves the desired security metrics (Fig. 3).

Supplementary Note 3

Machine learning simulation. Machine learning training on the MNIST and ECG datasets was carried out using the PyTorch framework, while explicitly embedding device-level behavior such as LTP/LTD characteristics, PUL responses, read-disturb effects, and conductance variations induced by conceal–reveal cycling of the V-NAND flash memory. As illustrated in Supplementary Fig. 1, we adopted a five-layer neural network architecture including the input layer. In the FF-based configuration, the network comprised four hidden layers, each with 500 neurons. In contrast, the BP-based network consisted of three hidden layers with 500 neurons per layer followed by a single output layer. Throughout all simulations, the ADC resolution was fixed to 7 bits.

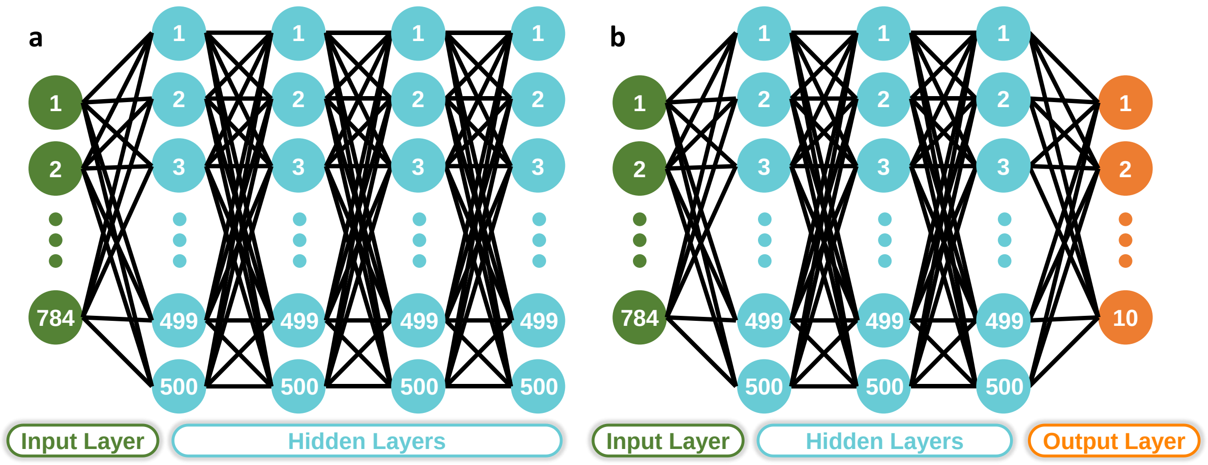

Supplementary Fig. 1 Neural network configuration for MNIST classification using **a** FF and **b** BP.

Supplementary Note 4

Implementation of model-inversion attack. To quantitatively evaluate the resistance of the proposed FF-PUNN to model-inversion (MI) attacks, we implemented a white-box activation-maximization attack on the trained neural networks. The attack takes as input a target class label *c* and attempts to synthesize an input image *x* whose forward-forward (FF) "goodness" strongly favors class *c* over the remaining classes.

For a given input vector *x* and target class *c*, we first define a class-dependent score using the same FF goodness used for training (see main manuscript). For each class label $k \in \{0,\ldots,9\}$, we form an input x_k by overwriting the first 10 elements of the flattened 28×28 image with a one-hot encoding of *k*, as in FF training. The network then performs a forward pass through all four hidden layers, and the layer-wise goodness is computed as $g_l(x_k) = \|y_l(x_k)\|_2^2$, where y_l is the ADC-quantized ReLU output of layer *l*. The total FF score for class *k* is the sum of these goodness values[3]: $S_k(x) = \sum_{l=1}^{4} g_l(x_k)$. Here, a higher $S_k(x)$ indicates that the hidden layers collectively regard *x* as a "good" example of class *k*, following the FF learning principle.

For a target class *c*, the MI attack maximizes the difference between the target score $S_c(x)$ and the average score of non-target classes $\bar{S}_{\neg c}(x)$, while regularizing the image with total-variation (TV) and l_2 penalties. The optimization objective is

$$L(x) = -\left(S_c(x) - \alpha \bar{S}_{\neg c}(x)\right) + \lambda_{TV} TV(x) + \lambda_{L2} \|x\|_2^2$$

, where $\bar{S}_{\neg c}(x) = \frac{1}{9} \sum_{k \neq c} S_k(x)$, α is a contrast weight, λ_{TV} controls spatial smoothness, and λ_{L2} penalizes large pixel values.

The TV term is computed on the 2D image *x* as

$$TV(x) = \frac{1}{N} \sum_{i,j} \left(|x_{i,j+1} - x_{i,j}| + |x_{i+1,j} - x_{i,j}| \right)$$

, where N is the number of pixels. In practice, we minimize L(x) with respect to x; maximizing the target-minus-non-target score corresponds to minimizing the first term.

Supplementary References

1. Yoo, H. N. et al. First Demonstration of 1-bit Erase in Vertical NAND Flash Memory. In 2022 *IEEE Symposium on VLSI Technology and Circuits* (eds Wong, H. S. P. & Kuroda, T.) 304–305 (IEEE, 2022).
2. Park, S. H., Koo, R. H., Yang, Y., Im, J., Ko, J. & Lee, J. H. Concealable physical unclonable functions using vertical NAND flash memory. *Nat. Commun.* **16**, 5155 (2025).
3. Hinton, G. The Forward-Forward Algorithm: Some Preliminary Investigations. Preprint at https://arxiv.org/abs/2212.13345 (2022).
